# ILeukin10Pred: A Computational Approach for Predicting IL-10-Inducing Immunosuppressive Peptides Using Combinations of Amino Acid Global Features

**DOI:** 10.3390/biology11010005

**Published:** 2021-12-21

**Authors:** Onkar Singh, Wen-Lian Hsu, Emily Chia-Yu Su

**Affiliations:** 1 Bioinformatics Program, Taiwan International Graduate Program, Institute of Information Science, Academia Sinica, Taipei 115, Taiwan; onkarnims2009@gmail.com (O.S.); hsu@iis.sinica.edu.tw (W.-L.H.); 2 Institute of Biomedical Informatics, National Yang Ming Chiao Tung University, Taipei 112, Taiwan; 3 Graduate Institute of Biomedical Informatics, College of Medical Science and Technology, Taipei Medical University, Taipei 110, Taiwan; 4 Department of Computer Science and Information Engineering, Asia University, Taichung 413, Taiwan; 5 Clinical Big Data Research Center, Taipei Medical University Hospital, Taipei 110, Taiwan

**Keywords:** interleukin-10, immunosuppressive peptides, machine learning, anti-inflammatory, cytokines, extra tree classifier

## Abstract

**Simple Summary:**

Interleukin-10 is a cytokine that exhibits potent anti-inflammatory characteristics that play an essential role in limiting the host’s immune response to pathogens and regulating the growth or differentiation of various immune cells. Moreover, interleukin-10 prediction via conventional approaches is time-consuming and labor-intensive. Hence, researchers are inclined towards an alternative approach to predict interleukin-10-inducing peptides. Additionally, numerous in silico tools are available to predict T cell epitopes. These methods generally follow a direct or indirect approach where they directly predict cytotoxic T-lymphocyte epitopes rather than major histocompatibility complex binders or indirectly predict single components of the T cell recognition pathway. However, very few studies are available that address cytokine-specific predictions. Our research utilized a computer-aided approach to develop a model to predict IL-10-inducing peptides. This study outperformed the existing state-of-the-art method and achieved an accuracy of 87.5% and Matthew’s correlation coefficient (MCC) of 0.755 on the hybrid feature types and outperformed an existing state-of-the-art method based on dipeptide compositions that achieved an accuracy of 81.24% and an MCC value of 0.59. Therefore, our model is promising to assist in predicting immunosuppressive peptides that induce interleukin-10 cytokines.

**Abstract:**

Interleukin (IL)-10 is a homodimer cytokine that plays a crucial role in suppressing inflammatory responses and regulating the growth or differentiation of various immune cells. However, the molecular mechanism of IL-10 regulation is only partially understood because its regulation is environment or cell type-specific. In this study, we developed a computational approach, ILeukin10Pred (interleukin-10 prediction), by employing amino acid sequence-based features to predict and identify potential immunosuppressive IL-10-inducing peptides. The dataset comprises 394 experimentally validated IL-10-inducing and 848 non-inducing peptides. Furthermore, we split the dataset into a training set (80%) and a test set (20%). To train and validate the model, we applied a stratified five-fold cross-validation method. The final model was later evaluated using the holdout set. An extra tree classifier (ETC)-based model achieved an accuracy of 87.5% and Matthew’s correlation coefficient (MCC) of 0.755 on the hybrid feature types. It outperformed an existing state-of-the-art method based on dipeptide compositions that achieved an accuracy of 81.24% and an MCC value of 0.59. Our experimental results showed that the combination of various features achieved better predictive performance..

## 1. Introduction

### 1.1. Roles of Interleukin (IL)-10 in Immune Responses

The complex network system of biological processes that protect organisms against infection is known as the immune system. This system comprises several crucial cell types, such as B cells, T cells, antigen-presenting cells (APCs), and the biochemical mediators that help communicate and relay signals. All these factors have essential roles in the immune system in defending the body against harmful substances. However, sometimes our immune system mistakenly attacks our own body and damages its tissue, causing autoimmune diseases. Recent advances suggested that deregulated function of the immune system contributes to the development of several disorders, including cancers and autoimmune diseases [1]. Almost 4% of the world’s population suffers from one of more than 80 distinct autoimmune diseases [2]. Immunosuppression-mediated anti-inflammatory cytokines can address the deregulated function of the immune system. These cytokines are an array of immunoregulatory molecules that limit the proinflammatory cytokine response, including interleukin (IL)-1 receptor a (1Ra), IL-4, IL-10, IL-11, IL-13, IL-33, IL-35, and IL-37 and transforming growth factor (TGF)-β.

IL-10 is a well-known and widely studied cytokine that Mossman and Coffman first discovered in 1988. This novel immune mediator is secreted by mouse type 2 T-helper (Th2) cell clones that inhibit the synthesis of IL-2 and interferon (IFN)-γ in Th1 cell clones, initially termed the cytokine synthesis inhibitory factor (CSIF), and later named IL-10 [3]. IL-10 is known for its essential pleiotropic immunoregulatory properties with multiple biological effects in different cell types [4]. This immunosuppressive cytokine is produced by several immune cells during the immune response, including monocytes, macrophages, Th2 cells, mast cells, natural killer (NK) cells, and a cluster of differentiation-positive (CD4+), CD25+, forkhead box p3 (Foxp3)+, and regulatory T (Treg) cells [5]. However, CD4+ cells are the principal producers among all mature T cells [6].

The IL-10 receptor (IL-10R) mediates the immunosuppressive activities of IL-10. This receptor comprises two subunits IL-10R1 and IL-10R2 that belong to the class II family of cytokine receptors (CRF2). The CRF2 family includes IL-19, IL-20, IL-22, IL-24/melanoma differentiation-associated gene (Mda)-7, IL-26, and interferons (IFN-α, -β, and -γ) [7]. The binding affinity between IL-10 and IL-10R is very high; however, this can vary among species. For example, mouse IL-10 can block the binding of human IL-10 to mouse but not human cells [8]. After binding occurs between IL-10 and IL-10R, the complex activates a Janus kinase (JAK)-signal transducer and activator of transcription (STAT) pathway to phosphorylate the receptors, creating docking sites for signaling molecules for STAT family members that are required for IL-10’s immunoregulatory effects [9].

IL-10 has a wide range of functions in hemopoietic cells, including well-known anti-inflammatory effects by macrophage deactivation and suppression of tumor necrosis factor (TNF)-α, IL-1β, and IL-6 [10]. Despite all the essential roles played by IL-10, certain chronic inflammatory diseases have been linked to overproduction or inappropriate production of IL-10, such as inflammatory bowel disease and several other autoimmune diseases [11]. Previous studies demonstrated that a deficiency in IL-10 induces autoimmune disease, including a wide range of experimental models, such as experimental autoimmune neuritis [12], systemic lupus erythematosus [13], experimental autoimmune encephalomyelitis [14], and rheumatoid arthritis [15]. Numerous clinical studies attempted to assess the efficacy of recombinant IL-10 in treating autoimmune diseases. Unfortunately, the results of most clinical trials did not meet expectations [16].

### 1.2. Literature Review

The fundamental step in applying bioinformatics and analytical methods to vaccine development is to determine and discriminate epitopes that are potentially immunoprotective from those that are not [17]. It is currently well known that epitopes link to MHC class I and II molecules in the binding groove by forming interactions between their R group side chains and pockets located on the floor of MHC [18,19]. This knowledge has led to several T cell epitope-mapping algorithms that rapidly identify putative T cell epitopes [20]. Tools developed during this period have one basic similarity: they focus on individual components of the T cell recognition pathway using either a direct or indirect approach [21]. Following the direct approach, the prediction tools directly predict cytotoxic T lymphocytes (an essential immune cell to identify antigens on cells and help eliminate them) instead of MHC binders, such as CTLPred [22] and NetCTLpan [23]. The CTLPred method is based on artificial neural network (ANN) and support vector machine (SVM) techniques and allows combined predictions using these two approaches. The NetCTLpan server is based on ANN techniques to predict CTL epitopes, which can be restricted to any MHC molecules of a known protein sequence.

MHC class I binder’s prediction tools follow the indirect approach. These tools focus only on one component instead of T cell epitopes, e.g., nHLApred [24], a hybrid approach to predict MHC class I-restricted T cell epitopes. ProPred1 is a matrix-based method to predict MHC-binding sites in an antigenic sequence for MHC class I alleles [25]. TAPPred is a prediction tool that predicts transporters associated with antigen processing (TAP)-binding peptides that remarkably help identify MHC class I-restricted T cell epitopes [26]. These tools are currently very efficient and have wide allelic coverage with high prediction accuracies. Notably, these methods predict T cell epitopes by following a direct or indirect approach but fail to provide information on the release of cytokines. To address this issue, Raghava’s group took into account and initiated the development of cytokine-specific prediction methods, for example, IL-4Pred [27], IL-10Pred [21], and IL-6Pred [28]. In this study, we developed a classification-based prediction method using a dataset (IL-10Pred) of the state-of-the-art method. This study involved various sequence-based compositional and physicochemical feature descriptors to identify and predict peptides that induce IL-10 and those that do not induce IL-10.

### 1.3. Challenges in Predicting IL-10-Inducing Peptides

Immune cells resist inflammation by producing anti-inflammatory cytokine, IL-10. However, cytokines have a short half-life in circulation, and their impacts on cell activities are limited. Consequently, IL-10 therapy using recombinant native IL-10 has had only limited success in treating human diseases [29] and have been shown to cause side effects. Moreover, IL-10 as a therapeutic tool was reported to cause side effects in several studies [30]. Contrarily, peptide-based epitopes that induce IL-10 may be a promising alternative [31]. However, there are numerous challenges during the development phase, including epitope identification, lack of immunogenicity, clinical evaluation, immune evasion, and many more. Additionally, these methods are labor-intensive with high manufacturing costs. The biggest challenge for developing in silico models for such predictions is the requirement for factual data that have been experimentally validated. In this study, we retrieved a dataset from the IL-10Pred state-of-the-art method derived from the Immune Epitope Database (IEDB) [32].

## 2. Materials and Methods

### 2.1. Dataset Acquisition and Preprocessing

To construct the interleukin-10 prediction model (ILeukin10Pred), we collected a benchmark dataset from a published article on IL-10Pred [21]. This dataset was derived from the IEDB, the largest repository of immune epitopes [32]. To build a positive dataset, all experimentally validated MHC II binders that trigger the release of IL-10 were extracted and denoted as IL-10-inducing peptides. Those MHC II binders that were not responsible for triggering IL-10 were assigned as non-IL-10-inducing peptides. The final dataset consisted of 394 IL-10-inducing and 848 non-inducing peptide sequences. Because the sequence numbers of positive (IL-10-inducing peptides) and negative instances were extremely imbalanced (the number of negative instances was almost double the number of positive instances), we used Azure Machine Learning Studio [33] to perform SMOTE (Synthetic Minority Oversampling Technique).

### 2.2. Feature Encoding

In the machine learning-based prediction method, the first critical step was amino acid sequence encoding, which plays a crucial role in the success of these methods. This study used six feature representation schemes of amino acid composition (AAC); dipeptide composition (DPC); composition, transition, and distribution (CTD); quasi-sequence order (QSO); sequence order coupling (SOC); and autocorrelation (AutoC). We used the BioTriangle web server [34], a comprehensive molecular representation platform that allows users to generate various molecular descriptors of proteins or peptides. All feature descriptors used in this study are briefly described here.

#### 2.2.1. Amino Acid Composition (AAC)

The AAC is defined as the frequency of each amino acid in a peptide or protein sequence [35]. The frequency of all naturally occurring amino acids can be calculated using Equation (1):

(1) $$f\;\left(i\right)=\frac{N\left(i\right)}{N},\;i\;\sum \left\{A,C,D,E,.\;.\;.,Y\right\}$$

where *N(i)* is the number of amino acid type *i*, and *N* is the length of the peptide or protein sequence.

#### 2.2.2. Dipeptide Composition (DPC)

The DPC is defined as the frequency of two amino acid types in a peptide or protein sequence [36]. This scheme generated a total of 400 feature descriptors. The DPC is defined in Equation (2):

(2) $$D_{\left(rs\right)}=\frac{N_{rs}}{N-1}\;r,s\;\sum \left\{A,C,D,E,.\;.\;.,Y\right\}$$

where *Nrs* is the number of dipeptides represented by amino acid types *r* and *s*.

#### 2.2.3. Composition, Transition, and Distribution (CTD)

The CTD feature descriptor represents the distribution of amino acid patterns for particular structural and physicochemical properties, such as hydrophobicity, normalized van der Waals volume, polarity, polarizability, charge, secondary structures, and solvent accessibility of the peptide or protein sequences [37,38,39,40]. Composition is defined as the number of amino acids of an individual property divided by the total number of amino acids. Transition measures the percentage frequency with which amino acids of another property follow amino acids of a specific property. Distribution measures the chain length within which the first 25%, 50%, 75%, and 100% of the amino acids of a distinct property are located.

#### 2.2.4. Quasi-Sequence Order (QSO)

QSO descriptors are derived from the distance metrics in between 20 amino acids [41], as proposed by Chou et al. [42]. For each amino acid type, type-1 QSO ($X_{r}$) can be defined as in Equation (3):

(3) $$X_{r}=\frac{f_{r}}{\sum_{r=1}^{20}f_{r}+w\sum_{d=1}^{maxlag}\tau_{d}}\;r=1,\;2\ldots ,20+maxlag$$

where *f_r_* is the normalized occurrence of amino acid type *i*, and *w* is a weight factor (*w* = 0.1) for the first 20 QSO descriptors. The type-2 QSO (*X_d_*) is defined as in Equation (4):

(4) $$X_{d}=\frac{w\tau_{d}-20}{\sum_{r=1}^{20}f_{r}+w\sum_{d=1}^{maxlag}\tau_{d}}\;d=21,22\ldots ,30+maxlag$$

#### 2.2.5. Sequence Order Coupling (SOC) Number

The *d*-th rank SOC number is defined by Equation (5):

(5) $$\tau_{d\;}\;d=\sum_{i-1}^{N-d}{\left(d_{i,i+d}\right)}^{2}\;d=1,2,3,.\;.\;.,\;nlag$$

#### 2.2.6. Autocorrelation (AutoC)

AutoC descriptors are defined based on the distribution of amino acid properties along the sequence. There are three different AutoC descriptors, viz., Geary, Moran, and Moreau-Broto. Moran and Geary AutoC descriptors can be expressed by Equations (6), (7), and (8), respectively:

(6) $$AC_{l}=\frac{\sum_{i=1}^{N-1}\;P_{i}P_{i+l}}{N-l}\;l=1,2,3,4\;$$

(7) $$MA_{l}=\frac{\frac{1}{N-l}\sum_{i-1}^{N-l}(P_{i}-\bar{P})\;(P_{i+1\;}-\bar{P})}{\frac{1}{N}\sum_{i=1}^{N}{\left(P_{i}-\bar{P}\right)}^{2}}\;l=1,\;2,\;3,\;4$$

(8) $$GA_{l\;}=\frac{\frac{1}{2\left(N-l\right)}\sum_{i=1}^{N-l}{\left(P_{i\;}-P_{i+l}\right)}^{2}}{\frac{1}{N-1}\sum_{i=1}^{N}{\left(P_{i}-\bar{P}\right)}^{2}}\;l=1,2,3,4$$

where *l* is the autocorrelation lag, *P_i_* and *P_i+l_* are the properties of amino acids at respective positions *i* and *i + l,* and $\bar{P}$ is the average value of property *P*,$\bar{\;P}$ = $\overset{N}{\underset{i=1}{\sum }}P_{i}/N$.

### 2.3. Imbalanced Dataset Handling by Oversampling

Once all features were generated, we normalized the data and used the synthetic minority oversampling technique to avoid biases toward the majority class (non-IL-10-inducing peptides). The SMOTE approach oversampled data of the minority class by generating synthetic data [43]. This approach is widely used for imbalanced datasets. This method is based on the nearest neighbors and predicted by Euclidean distances between data points in the feature space. In our study, we used 100% oversampling, which means that the number of synthetic samples created is always a multiple of 100. Hence, the number of minority classes was doubled. For each minority instance, a k number of nearest neighbors was found to belong to the same class:

(9) K=SMOTE%/100

Differences between the feature vector of the considered instance and feature vectors of the *k*-nearest neighbors were found. So, the *k* number of difference vectors was obtained. The *k* difference vectors were multiplied by a random number between 0 and 1 (excluding 0 and 1). After being multiplied by the random numbers, the difference vectors were added to the considered instance (original minority instance) feature vector at each iteration. The dataset after applying the SMOTE method is shown in Figure S1.

### 2.4. Machine Learning Algorithms

Classification is a crucial issue in data mining. For a given database of records, each assigned with a class label, a classifier generates a concrete and comprehensive overview of each class that can be used for further classification [44]. Nowadays, many classifiers with different virtues are used for binary classification tasks. In this study, we used PyCaret, a newly developed open-source, low-code, python-based machine learning library that allows users to evaluate the performances of various machine learning models together. This library comprises 15 different machine learning classifiers, such as extra trees classifier (ETC), extreme gradient boosting (XGB), light gradient boosting machine (LGBM), random forest (RF), ada boost classifier (ABC), logistic regression (LR), SVM-linear kernel, naive Bayes (NB), decision tree (DT), ridge classifier, K-nearest neighbor (KNN) classifier, quadratic discriminant analysis (QDA), linear discriminant analysis (LDA) CatBoost classifier, and gradient boosting classifier (GBC). At first, we implemented all machine learning models together to train and evaluate the model. Further, we optimized the accuracy metric to sort the top 3 models from the highest accuracy to the lowest accuracy. The top 3 models are briefly described here. ETC is an ensemble machine learning model that generates many unpruned decision trees from training datasets and uses major voting from decision trees for classification [45]. CatBoost stands for categorical boosting, and is an algorithm for gradient boosting on decision trees [46]. LGBM is another gradient boosting framework based on a decision tree that enhances the model’s efficiency with minimal memory usage [47].

### 2.5. Computational Framework

**Figure 1:**
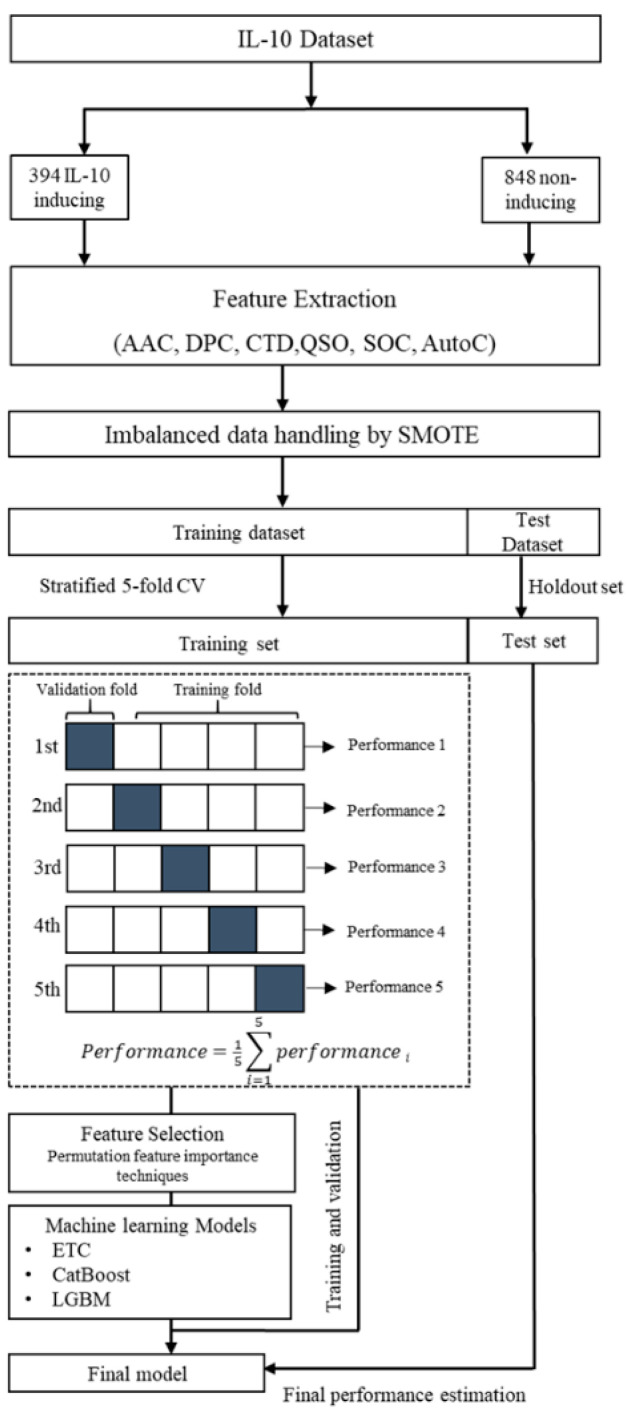
The systematic architecture of the proposed method, Ileukin10Pred, which included dataset collection, feature generation, SMOTE, feature selection, machine learning algorithms, and an evaluation process.

The computational framework of our proposed method for predicting IL-10-inducing peptides is illustrated in Figure 1. The analytical workflow comprised various steps, including dataset collection from the IL10Pred server [22], feature extraction, imbalanced data handling, feature selection, application of machine learning algorithms, and model evaluation. The first step was to download the IL-10 dataset from the state-of-the-art method. In the following steps, we used the BioTriangle webserver to extract and encode six sequence-based compositional and physicochemical features [34]. Moreover, the SMOTE method was applied to balance the dataset.

The dataset was further divided into 80% training data and 20% test data. We then trained and validated the model using stratified five-fold cross-validation. Additionally, a ‘classic’ feature selection method was applied that uses permutation feature importance techniques to select important discriminative biological features to feed into the machine learning models. The final model was evaluated on the holdout set. The holdout set was solely used for a performance assessment of our model; it was never used at any stage of model building or feature selection. Our system architecture represents the systematic procedures followed in this study. The name of our proposed method is ILeukin10Pred.

### 2.6. Performance Evaluation

Evaluating a model’s performance plays a crucial role in predictive modeling. Hence, selecting proper evaluation matrices is vital for any machine learning model. Here, we used various popular statistical measures to evaluate model predictions, such as the area under the receiver operating characteristics (ROC) curve (AUC), precision–recall (PR) curve, precision, sensitivity (Sen.)/recall, specificity (Spe.), accuracy (Acc.), and the Matthews correlation coefficient (MCC). The AUC is a widely accepted evaluation metric for classification-based machine learning models. The ROC curve is created by plotting sensitivity against [1-specificity]. This metric shows the ability of a model to discriminate the classes. The higher the AUC is, the better the prediction model is. Like the ROC curve, PR is used for evaluating the performance of binary classification algorithms when the classes are imbalanced, and the precision indicates how precise the positive predictions are. The higher the precision is, the lower the false positives are and vice versa, as shown in Equation (10), while the recall or sensitivity measures the number of correctly predicted positive outcomes from all true positives. The higher the recall is, the lower the false negatives are and vice versa as shown in Equation (11). Another way to calculate the PR curve is by finding the average precision (AP) as shown in Equation (12), where *P_n_* is the precision and *R_n_* is the recall at the nth threshold. Specificity, as represented in Equation (13), measures a negative outcome as positive (a false positive) out of the total true negatives. Accuracy is defined as the percentage of true predictions for the test data and can be calculated by Equation (14), with the true positives being divided by the total number of predictions. Last, we used one of the most reliable statistical rates, MCC, as shown in Equation (15). Based on the proportion of each class in its formula, its score is high only if the classifier performs well for both negative and positive elements:

(10) $$\mathrm{Precision}=\frac{TP}{TP+FP}\;$$

(11) $$\mathrm{Sen}.=\mathrm{Recall}=\frac{TP}{TP+FN}$$

(12) $$\mathrm{AP}=\sum (R_{n}-R_{n-1})P_{n}$$

(13) $$\mathrm{Spe}.=\frac{TN}{TN+FP}$$

(14) $$\mathrm{Acc}.=\frac{TP+TN}{TP+TN+FP+FN}$$

(15) $$\mathrm{MCC}=\frac{TP\times TN-FP\times FN}{\sqrt{\left(TP+FP\right)\left(TP+FN\right)\left(TN+FP\right)\left(TN+FN\right)}}$$

## 3. Results

### 3.1. Hyper-Parameters Tuning

Our machine learning model for IL-10 peptide prediction consisted of multiple hyper-parameters. To tune the hyper-parameters, we performed stratified 5-fold cross-validation. The hyper-parameters were tuned using a random grid search method. The parameters of our best models are mentioned as follows:

#### 3.1.1. ET Classifier

*n* estimators = 200, criterion = ‘gini’, max depth = 6, min samples split = 7, min samplesleaf = 4, min weight fraction leaf = 0.0, max features = 1.0, max leaf nodes = None, min impurity decrease = 0.0, bootstrap = False, oob score = False, *n* jobs = −1, random state = 123, verbose = 0, warm start = False, class weight = balanced subsample, ccp alpha = 0.0, max samples = None.

#### 3.1.2. LGBM Classifier

Boosting type = gbdt, class weight = None, colsample bytree = 1.0, importance type = split, learning rate = 0.1, max depth = −1, min child samples = 20, min child weight = 0.001, min split gain = 0.0, *n* estimators = 100, *n* jobs = −1, num leaves = 31, objective = None, random state = 123, reg alpha = 0.0, reg lambda = 0.0, silent = True, subsample = 1.0, subsample for bin = 200,000, subsample freq = 0.

#### 3.1.3. CatBoost Classifier

Nan mode = Min, eval metric = Logloss, iterations = 1000, sampling frequency = Per Tree, leaf estimation method = Newton, grow policy = SymmetricTree, penalties coefficient = 1, boosting type = Plain, model shrink mode = Constant, feature border type = GreedyLogSum, bayesian matrix reg = 0.10, l2 leaf reg = 3, random strength = 1, rsm = 1, boost from average = False, model size reg = 0.5, pool metainfo options={‘tags’:{}}, subsample = 0.8, use best model = False, class names = [0, 1], random seed = 123, depth = 6, posterior sampling = False, border count = 254, classes count = 0, auto class weights = None, sparse features conflict fraction = 0, leaf estimation backtracking = AnyImprovement, best model min trees = 1, model shrink rate = 0, min data in leaf = 1,loss function = Logloss, learning rate = 0.01, score function = Cosine, task type = CPU, leaf estimation iterations = 10, bootstrap type = MVS, max leaves = 64.

### 3.2. Analysis of Amino Acid Position Preferences

In this section, we investigated the position preference of amino acid residues for both IL-10-inducing peptides and non-IL-10-inducing peptides. Here, we considered two crucial physicochemical properties (charge and hydrophobicity) of the amino acid residues that play vital roles in anti-inflammatory peptides. Furthermore, Ialenti et al. reported that the anti-inflammatory activity of a molecule is in the N-terminal region [48]. As a proof of concept, we further investigated the position preference of amino acids with the help of a two-sample logo (TSL). The height of the peptide logo was scaled (t-test by *p* < 0.05) for statistical significance. For the preference of amino acid residues at both the N and C terminals, we considered the average peptide length (i.e., 16 amino acid residues) of IL-10-inducing and non-IL-10-inducing peptides. The first eight residues represent the N-terminal, and the last eight residues represent the C-terminal.

**Figure 2:**
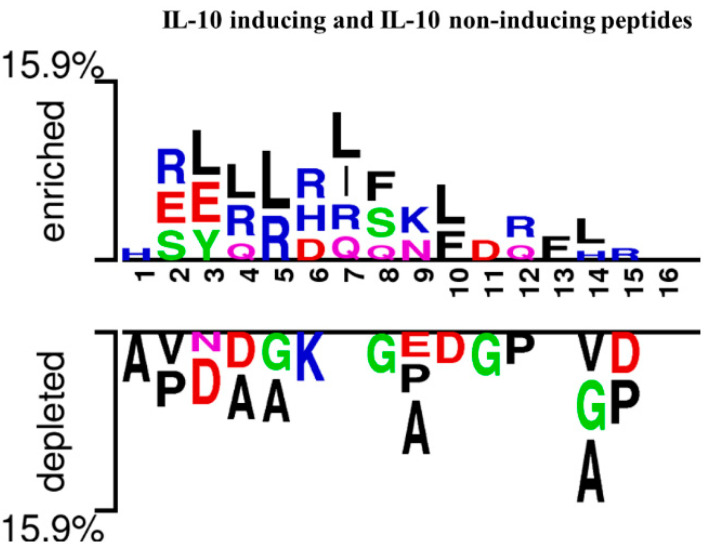
Two-sample logo showing the preference of positively charged and hydrophobic residues in IL-10-inducing peptides and non-IL-10-inducing peptides at different positions. The first eight positions represent the N-terminus of peptides, and the last eight positions represent the C-terminus of peptides.

In Figure 2, we show the position preferences of positively and negatively charged amino acid residues for both datasets. Our analysis revealed that the preferences of positively charged residues were abundantly present in IL-10-inducing peptides like histidine (H) at the 1st position, arginine (R) at the 2nd and 6th positions of the N-terminal, and lysine (K) at the 9th, and arginine at the 12th and 15th positions of the C-terminal. Conversely, for non-IL-10-inducing peptides, a negatively charged residue (aspartic acid, D) was present at the 3rd position of the N-terminal and 10th position of the C-terminal. Additionally, we made other observations based on hydrophobicity, and we found that leucine (L) frequently appeared at the 3rd, 4th, 5th, 7th, 10th, and 14th positions and phenylalanine at the 8th and 13th positions. However, for non-IL-10-inducing peptides, alanine (A) was more predominant at the 1st, 4th, and 5th positions of the N-terminal and 9th and 14th positions of the C-terminal.

### 3.3. Compositional Analysis of IL-10 Datasets

**Figure 3:**
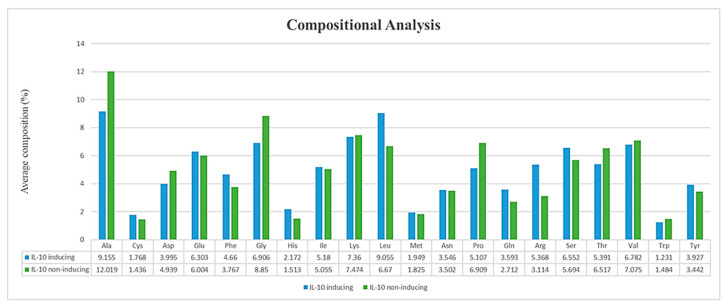
Average percentages of amino acid compositions (AACs) in IL-10-inducing peptides and non-IL-10-inducing peptides.

The composition of amino acids of any peptide defines its function and quality. Here, we conducted a composition analysis of IL-10-inducing and non-IL-10-inducing peptides. The average compositions of amino acids for both datasets are shown in the bar plot of Figure 3. In terms of hydrophobic and positively charged residues, the compositional analysis revealed that among 20 amino acids, 12 of them were dominant in IL-10-inducing peptides, in which positively charged residues were arginine (R) and histidine (H), and hydrophobic residues were isoleucine (I), leucine (L), methionine (M), phenylalanine (F), and tyrosine (Y). In contrast, in non-IL-10-inducing peptides, hydrophobic residues of alanine (A), valine (V), and tryptophan (W) were dominant, whereas lysine (K) was the only dominant positively charged residue. Interestingly, the amino acids found in IL-10-inducing peptides had great significance in terms of immune responses.

### 3.4. Machine Learning Model Prediction of Single Feature Types

Each biological feature in our experiment was evaluated separately. Here, we used six distinct (i.e., AAC, DPC, CTD, QSO, SOC, and AutoC) sequence, structure, and physicochemical features to delineate their roles in distinguishing IL-10-inducing and non-IL-10-inducing peptides. We performed stratified five-fold cross-validation on the training dataset to fit and evaluate the model multiple times. For feature selection, we applied the ‘classic’-based feature selection method. Further, the performance of our model was assessed using a holdout/test set that was never used at any stage of model building or feature selection, so the performance was evaluated only one time. The predictive performance of the top three models (ETC, CatBoost, and LGBM) for the training with their means ± standard deviations (SDs), and test dataset results are shown in Table 1. We compared the accuracy, AUC, and MCC values of different algorithms based on the above-mentioned features. The experimental results showed that the AAC descriptors of the LGBM classifier achieved the highest accuracy of 85.4%, with AUC and MCC values of 0.903 and 0.712, respectively.

Additionally, to check the importance of selected feature types, we performed experiments on unselected or junk features previously discarded from the models. We observed that performance results on both training and test data drastically decreased for single feature types, particularly ACC and CTD. The performance results of all models on single feature types are mentioned in the Table S1.

Furthermore, analyzing the roles amino acids play in biological systems is one way of evaluating the effectiveness of an amino acid-encoding scheme. Using this strategy, we interpreted the model based on SHapley Additive exPlanations (SHAP) to observe the predictions generated from our trained model. We determined each feature’s impact on the model output through this representation by examining a density scatterplot of SHAP values for each feature in the test dataset. All features were sorted by the sum of SHAP values across all samples. In Figure S2a, for AAC, we observed that R, G, V, F, and L amino acid residues had a better impact than other amino acid residues. Notably, some of these residues were dominant in our position preference and composition analysis (Figure 2 and Figure 3). ETC achieved the highest accuracy of 86.6% for DPC descriptors, with an AUC of 0.943 and an MCC value of 0.733. One possible reason for the DPC achieving a higher prediction performance was due to the information implicit in dipeptides, as it contains more structure and sequence information than the AAC. Moreover, in a recent publication by Wang et al., out of 18 dipeptides derived from ovotransferrin, five dipeptides (FL, LL MK, HC, and CR) were shown to increase IL-10 gene expression along with inhibiting proinflammatory cytokines [49].

Similarly, dipeptides predicted by our model also supported the above study, as dipeptides FF and LL had the highest impacts on the model performance (Figure S2b). CTD descriptors were based on the overall composition, transition, and distribution of amino acid attributes, such as the secondary structure, solvent accessibility, hydrophobicity, charge, normalized van der Waals volume, polarity, and polarizability [50]. The charge, van der Waals, and secondary structure showed the highest impacts on model outputs, with an accuracy of 83.8%, an AUC of 0.913, and an MCC value of 0.678 by ETC (Additional Figure S2c). Conversely, the CatBoost classifier outperformed other ML models on AutoC descriptors, which were based on the distributions of various amino acid properties along with the sequence. As shown in Figure S2d, stericity, hydrophobicity, and mutability had the highest impacts on the model output and achieved an accuracy of 85.9%, an AUC of 0.909, and an MCC of 0.719. Sequence orders had two attributes of SOC and QSO. CatBoost attained the highest accuracy of 89.9% for SOC descriptors, with an AUC of 0.936 and an MCC value of 0.801. For the QSO descriptor, ETC achieved the optimum performance with an accuracy of 86.3%, an AUC value of 0.924, and an MCC value of 0.726. The sequence order features represent amino acid distribution patterns corresponding to specific physicochemical properties along with a protein or peptide sequence [51]. The best performing features of SOC and QSO are illustrated in Figure S2e,f.

### 3.5. Machine Learning Predictions of Hybrid Feature Types

In our study, the predictive performance based on hybrid features was investigated. Hybrid features denote combinations of sequence and physicochemical features. These data could be enriched with information derived from combinations of such features. This section combines all feature types to explore the impacts of the biological properties in predicting IL-10-inducing peptides. For feature selection, we performed a classic-based feature selection method using permutation feature importance techniques with a feature selection threshold of 0.9, and we obtained 1342 selected features.

**Figure 4:**
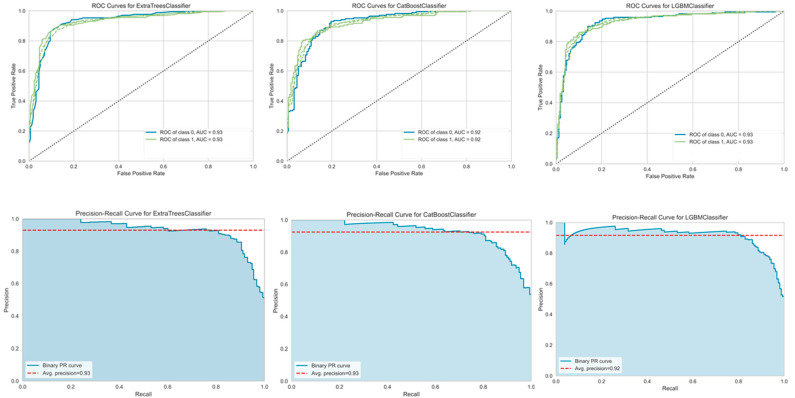
The area under the receiver operating characteristics (AUC) curve and the area under the precision–recall (AUCPR) curve show model performances, developed using selected features on the holdout set.

Various machine learning algorithms were employed on the selected feature sets, and the holdout set was used to evaluate the predictive performances of the best three models. Among the best three predictive models, the ETC achieved an 86.5% accuracy and an AUC of 0.929 on the training dataset, whereas, on the test dataset, it achieved an 87.50% accuracy, a 0.931 AUC value, and an MCC of 0.755 with a PR curve of 0.93. We list the other classifier’s predictive performances on the benchmark training and test datasets in Table 2, and the AUC and PR curve values are shown in Figure 4. This figure provides a graphical representation of a classifier’s performance for the top three highest performing classifiers, i.e., the ETC, CatBoost, and LGBM. The ETC outperformed the other classifiers used in this study. The ROC curve was generated by calculating and plotting the true positive rate against the false positive rate, and the higher the AUC score was, the better the classifier performance was. Similarly, we also provided a graphical representation of the PR curve. This was generated by calculating and plotting the precision against the recall. The PR curve can be calculated by finding the AP score. The higher the PR curve score was, the better a classifier performed.

Additionally, we also explored five other combinations, such as AAC+DPC, CTD+AutoC, QSO+SOC, AAC+DPC+CTD, and QSO+SOC+AutoC, where the ETC model on the AAC+DPC hybrid feature outperformed all the models with a performance of 87.2% accuracy and 0.946 AUC and an MCC value of 0.751. All model performances based on other combinations are mentioned in Table S2. Moreover, we carried out experiments on unselected or junk features previously discarded from models to determine the importance of the selected feature types. As a result, the models’ performance both on the test and training data shows a 4–5% performance reduction in comparison to the models’ performance on the optimal features. The performance results on the unselected feature sets are mentioned in Table S3.

## 4. Discussion

Applying bioinformatics to vaccine development has an essential role in deciphering molecular characterizations of infectious pathogens. At the same time, to combat the debilitating disease agent, severe acute respiratory syndrome coronavirus 2 (SARS-CoV-2), bioinformatics applications have experienced unprecedented growth during the recent outbreak. In silico methods of bioinformatics, immune-informatics, and so forth can be used for more rapid and precise vaccine designs. Another great aspect of using a computational approach for prediction analyses is that it can effectively reduce labor-intensive and time-consuming work compared to conventional approaches. Therefore, in this study, we developed a sequence-based computational method for determining IL-10-inducing peptides.

### 4.1. Position Preference and Composition Analysis of Amino Acids

We performed a composition and position preference analysis between IL-10-inducing and non-IL-10-inducing peptides to understand the residue preference using a benchmark dataset. Since IL-10 exhibits potent anti-inflammatory properties, this property can be influenced by hydrophobic amino acid residues and positively charged residues. Therefore, in this analysis, we mainly focused on two types of residues (hydrophobic and positive charged residues) to determine IL-10-inducing peptides. In both analyses, we observed that hydrophobic (I, L, M, F, and Y) and positively charged residues (R and H) frequently occurred in IL-10-inducing peptides, whereas three hydrophobic residues (A, V, and W) and only one positively charged residue (K) were dominant in non-IL-10-inducing peptides. Intriguingly, a study by Gesser et al. reported the N-terminal and C-terminal residues when comparing the homology of IL-10 sequences among humans, mice, and viral IL-10. They found that A and Y were part of the N-terminal region, whereas I, L, M, and K were present in the C-terminal region [52].

Second, the composition analysis revealed some crucial amino acids that exhibit anti-inflammatory properties and play crucial roles in immune responses, such as arginine, the primary function of which is to regulate cytokine production and eliminate pathogens. Researchers, using in vitro studies, determined that the presence of certain amounts of arginine was necessary for the maximal proliferation of rodent and human T lymphocytes upon exposure to mitogens and the killing of tumor cells by activated macrophages [53]. However, a recent publication reported that inflammatory macrophages utilize arginine for nitric oxide (NO). As a result, IL-10 can reduce NO production by inhibiting the expression of the messenger (m)RNA of inducible NO synthase (iNOS) or by enhancing its degradation [54]. Cysteine regulates the cellular redox state, and a study of the IL-10 crystal structure revealed that IL-10 is stabilized by two intramolecular disulfide bridges, Cys12–Cys108 and Cys62–Cys114. [55]. Histidine, one of the most common naturally occurring amino acids, was reported to act as a radical scavenger during epithelial injury. A recent study on an IL-10(-/-) transfer model of colitis stated that dietary histidine reduced histologic damage and colon weight, and tumor necrosis factor (TNF)-α mRNA expression and histidine inhibited lipopolysaccharide (LPS)-induced nuclear factor (NF)-κB in macrophages [56]. Other amino acids, such as leucine, also regulate the immune response while lysine possesses antiviral activity and regulates NO synthesis.

### 4.2. Biological Assessment of the Top 10 Selected Features

**Figure 5:**
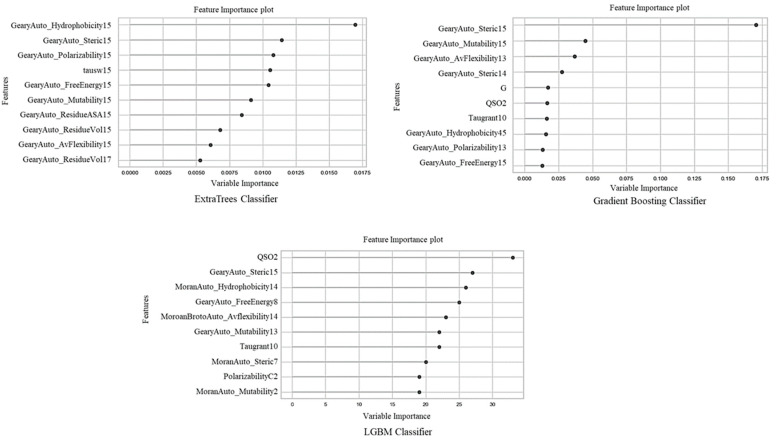
A plot of the top 10 important features for the IL-10 training datasets.

Feature selection becomes essentially predominant when a dataset consists of several features. In this study, we used a ‘classic’ method based on the permutation feature importance technique to identify important subsets of features for a given classification task. These methods can be used to identify and eliminate unneeded, irrelevant, and redundant attributes from data that might affect a predictive model’s accuracy, and this enables machine learning algorithms to train faster. We show the top 10 selected feature importance plots in Figure 5.

These top 10 descriptors were distribution features of amino acids with certain physicochemical properties, such as hydrophobicity, charge, mutability, residue’s solvent accessibility, polarizability, Moran and Geary AutoC, and QSO. Interestingly, we observed that among these selected features, some features fell into the category that may play a crucial role during the formation of the IL-10/IL-10R1 complex, such as hydrophobicity, charge, and a residue’s solvent accessibility. A recent study investigated the roles of surface charge and hydrophobicity distribution of the IL-10-binding interface. They found a significant difference in surface charge distributions at the interface between IL-10 and IL-10Ra, suggesting that electrostatic interactions might primarily determine IL-10 binding to IL-10Ra, where IL-10 has a more positively charged binding region. Similarly, in our amino acid composition analysis, we observed the preference for positively charged amino acids in IL-10-inducing peptides.

In terms of mutability, various studies reported the effects of mutations on IL-10R. Those reports suggested that a mutation in the *IL-10R* subunit gene was linked to early-onset enterocolitis, which involves hyperinflammatory immune responses in the gut [57]. Measurement of residues in proteins is based on their relative solvent accessibility (RSA), which describes how much of that residue is exposed in the tertiary structure. RSA is frequently used to describe a protein’s biophysical or evolutionary characteristics [58]. However, very few studies have reported the aforementioned features, that requires further investigation to decipher the importance of amino acid physicochemical properties either in inducing IL-10 or influencing its anti-inflammatory properties.

### 4.3. Evaluation of Single-Feature Types versus Hybrid-Feature Types

This study used two approaches to understand the importance of feature descriptors in identifying IL-10-inducing peptides, i.e., single-feature types and hybrid-feature types. We computed all six features individually for the positive and negative datasets to train and test a single-feature-type model. SOC and DPC achieved the highest accuracies, followed by QSO, AutoC, AAC, and CTD. The results suggested that both SOC and DPC features could be better indicators for predicting IL-10-inducing peptides. Similarly, we made another attempt to develop a prediction model by merging all feature types. The ETC outperformed the other models used in this study. When developing our model based on the hybrid-features set, we observed that the physicochemical properties of the Geary AutoC, such as the hydrophobicity scale, average flexibility index, polarizability parameter, free energy of a solution in water, accessible surface areas, residue volume, steric parameters, and relative mutability, were prominent and frequently occurred in the top 10 selected features. In contrast, the DPC and SOC feature individually performed better, but in the case of hybrid features, the DPC feature variable importance was less than the other feature types and failed to land in the list of top 10 features shown in Figure 5. This analysis revealed the importance of hybrid features over single-feature types.

### 4.4. Performance Comparison with the State-of-the-Art Method

Using the benchmark dataset, we compared our Ileukin10Pred method with the state-of-the-art IL-10-inducing peptide prediction method (IL-10Pred). As shown in Table 2, our ETC model attained the best performance with an 87.5% accuracy, an AUC of 0.931, and an MCC of 0.755 on the holdout set. Moreover, LGBM also achieved a better performance than the state-of-the-art method in terms of an accuracy of 87.2%, an AUC of 0.929, and an MCC of 0.747, whereas CatBoost achieved an accuracy of 86.6%, an AUC of 0.923, and an MCC value of 0.737. In this study, we used various approaches to examine the performance of our model. We first developed our model on the benchmark IL-10 training dataset and attained the best performance among other classifiers used in this study.

This study focused on understanding correlations between amino acid sequence features and IL-10-inducing peptides. Since it is well-known that amino acids are building blocks of proteins, and even though they all share a carboxyl group, an amine group, and a side chain, the various functional groups in the side chain often contribute to the distinctive properties affect the formation and function of proteins. Hence, it is crucial to consider various distinctive properties of amino acids to develop a more precise prediction model. Similarly, when compared to the state-of-the-art method, the model was developed by utilizing a single-feature type (DPC) only.

Conversely, this study was not only restricted to single-feature types. Besides, we first investigated all features individually and observed the prediction performances of all models. After that, we combined all features and used them as a hybrid of all features to further develop our model. By doing this, we not only achieved better prediction performances but were also able to determine the most important features that contributed to IL-10-inducing peptide predictions. However, further experimental studies are required to elucidate the importance of the physicochemical properties of amino acids in identifying IL-10-inducing immunosuppressive peptides.

### 4.5. Advantages and Limitations of ILeukin10Pred

This section summarizes a theoretical comparison of the proposed ILeukin10Pred with the existing state-of-the-art method. ILeukin10Pred investigated primary sequence-based, physicochemical-based, and evolutionary features. However, in IL-10Pred [22], only primary sequence encoding schemes were investigated. For instance, the IL-10pred method analyzed primary sequence-based descriptors, such as AAC, DPC, and the sequence-based binary profile. Conversely, in this study, we did not restrict ourselves to investigating only primary sequence-based features. Instead, we also included and investigated physicochemical-based and evolutionary features individually and a combination of all these features (hybrid types) to examine their importance in predicting IL-10-inducing peptides. Here, we observed that hybrid-based features outperformed single-based feature types in predicting IL-10-inducing peptides. A limitation of our study is that the dataset used was comparatively small. Notably, it is imperative to consider the quantity and quality of the data when developing more accurate and reliable methods. Including more data for the peptide prediction task would reveal more insights, as the data provide information instead of relying on assumptions and weak correlations. Another possibility to further improve the prediction performance is to use blended models, where multiple models were combined to predict IL-10-inducing peptides.

### 4.6. Future Work

In this work, we used a sequence-based feature to develop a classification-based prediction method for IL-10-inducing peptides using a small dataset with a state-of-the-art method. For future work, we plan to add more experimentally verified data to the training set and investigate only structural features of the peptides that might provide deeper insights into understanding the molecular phenomenon of IL-10. Additionally, we plan to build our next prediction model using blended models, which might increase the performance and robustness of the model.

## 5. Conclusions

IL-10 is a pleomorphic cytokine that exhibits a broad spectrum of pleiotropic effects in immune regulation and inflammation, initially discovered as a product of Th2 cells that inhibit the production of Th1. However, later it was shown to be produced by various types of cells, including monocytes, macrophages, Th2 cells, mast cells, NK cells, and CD4+, CD25+, and Foxp3+ Tregs. Potent anti-inflammatory characteristics of IL-10 play a central role in maintaining normal tissue homeostasis. Similarly, defective IL-10 can lead to the development of autoimmune diseases. Nowadays, immunosuppressive medication is the core of conventional therapies for autoimmune diseases. These agents show promising results and remain the “gold standard” of care. However, prolonged use of these medications can result in toxicity and severe side effects in some patients. Thus, there is an imperative need to develop more specific strategies that might improve tolerability while minimizing adverse effects [59].

To address these concerns, several studies administered peptides that induce IL-10, which showed great potential over the direct use of IL-10 [60]. Hence, predicting IL-10-inducing peptides has become crucial for subunit vaccine design. To date, numerous computational methods have been developed to predict T cell epitopes; however, only one approach exists to address predictions of IL-10-inducing peptides (IL-10Pred). In this study, we developed a model (ILeukin-10Pred) to predict IL-10-inducing peptides, utilizing amino acid compositions and physicochemical-based features.

## Figures and Tables

**Table 1:** Performances of machine learning models based on single-feature types for the benchmark training and test datasets. Values shown are the mean ± standard deviation for the training dataset.

Training Set									
Feature	ETC			CatBoost			LGBM		
	Acc. %	AUC	MCC	Acc. %	AUC	MCC	Acc. %	AUC	MCC
AAC	82.3 ± 0.022	0.906 ± 0.013	0.647 ± 0.046	86.1 ± 0.022	0.920 ± 0.018	0.722 ± 0.045	85.3 ± 0.017	0.919 ± 0.014	0.707 ± 0.035
DPC	86.5 ± 0.008	0.942 ± 0.004	0.730 ± 0.017	84.2 ± 0.010	0.922 ± 0.011	0.685 ± 0.021	85.4 ± 0.015	0.920 ± 0.012	0.709 ± 0.029
CTD	84.6 ± 0.014	0.915 ± 0.005	0.693 ± 0.027	85.4 ± 0.013	0.912 ± 0.010	0.708 ± 0.025	85.6 ± 0.023	0.913 ± 0.012	0.704 ± 0.046
AutoC	82.9 ± 0.021	0.905 ± 0.012	0.664 ± 0.042	84.9 ± 0.014	0.903 ± 0.009	0.699 ± 0.029	84.8 ± 0.015	0.907 ± 0.014	0.696 ± 0.029
QSO	86.8 ± 0.013	0.925 ± 0.009	0.7369 ± 0.025	84.8 ± 0.029	0.912 ± 0.016	0.695 ± 0.055	84.3 ± 0.021	0.911 ± 0.019	0.687 ± 0.041
SOC	82.3 ± 0.004	0.887 ± 0.005	0.649 ± 0.008	80.7 ± 0.014	0.876 ± 0.008	0.619 ± 0.027	80.4 ± 0.016	0.869 ± 0.014	0.608 ± 0.033
**Test Set**									
**Feature**	**ETC**			**CatBoost**			**LGBM**		
	**Acc. %**	**AUC**	**MCC**	**Acc. %**	**AUC**	**MCC**	**Acc. %**	**AUC**	**MCC**
AAC	83.5	0.912	0.674	85.1	0.919	0.705	85.4	0.903	0.712
DPC	86.6	0.943	0.733	84.5	0.925	0.689	84.8	0.919	0.695
CTD	83.8	0.913	0.678	83.8	0.887	0.677	83.8	0.891	0.677
AutoC	84.8	0.922	0.698	85.9	0.909	0.719	84.8	0.916	0.695
QSO	86.3	0.924	0.726	82.9	0.906	0.658	86.3	0.910	0.725
SOC	87.8	0.952	0.757	89.9	0.936	0.801	86.9	0.932	0.737

**Table 2:** Performances and comparison with state-of-the-art machine learning models based on hybrid features for the benchmark training and test datasets. The values shown are mean ± standard deviation for the training dataset.

Training Set						
Model	Acc. (%)	AUC	Recall/Sen. (%)	Specificity (%)	Precision (%)	MCC
ETC	86.5 ± 0.013	0.929 ± 0.015	82.2 ± 0.004	89.8 ± 0.025	88.3 ± 0.025	0.724 ± 0.027
LGBM	86.3 ± 0.015	0.918 ± 0.013	83.8 ± 0.016	88.6 ± 0.029	87.3 ± 0.025	0.726 ± 0.030
CatBoost	86.2 ± 0.019	0.916 ± 0.019	83.1 ± 0.009	88.9 ± 0.034	87.6 ± 0.033	0.724 ± 0.039
**Test Set**						
**Model**	**Acc. (%)**	**AUC**	**Recall/Sen. (%)**	**Specificity (%)**	**Precision (%)**	**MCC**
IL-10Pred	81.2	0.880	79.7	81.9	N/A *	0.590
ETC	87.5	0.931	80.4	94.7	92.7	0.755
LGBM	87.2	0.929	81.0	91.7	91.4	0.747
CatBoost	86.6	0.923	79.1	92.9	91.9	0.737

* N/A denotes “not available.” The precision score of IL-10Pred is not available in the manuscript (Nagpal et al., *Scientific Reports*, 2017).

## Data Availability

All data generated or analyzed during this study are included in the supplementary information. The codes and dataset used in this study are publicly available at https://github.com/onkarS23/ ILeukin10Pred, accessed on 20 October 2021.

## Supplementary Materials

The following are available online at https://www.mdpi.com/article/10.3390/biology11010005/s1, Figure S1: Dataset ratio representation before and after applying synthetic minority oversampling technique (SMOTE). Figure S2: Model interpretation based on SHapley Additive exPlanations (SHAP) for single feature types. Table S1: Performances of machine learning models based on unselected features of all single-feature types for the benchmark training and test datasets. Table S2: Performances based on various hybrid feature combinations on the benchmark training and test datasets. Table S3: Performances of machine learning models based on unselected features on the hybrid dataset (Combinations of all feature types) for the benchmark training and test datasets.
