# Supplementary material for: ILeukin10Pred: A Computational Approach for Predicting IL-10-Inducing Immunosuppressive Peptides Using Combinations of Amino Acid Global Features
Source: Biology (Basel). 2021 Dec 21;11(1):5. doi: 10.3390/biology11010005 (PMC8773200; doi:10.3390/biology11010005)
Supplement: Supplementary file 1 [file biology-11-00005-s001.zip › Figure S1.pdf]

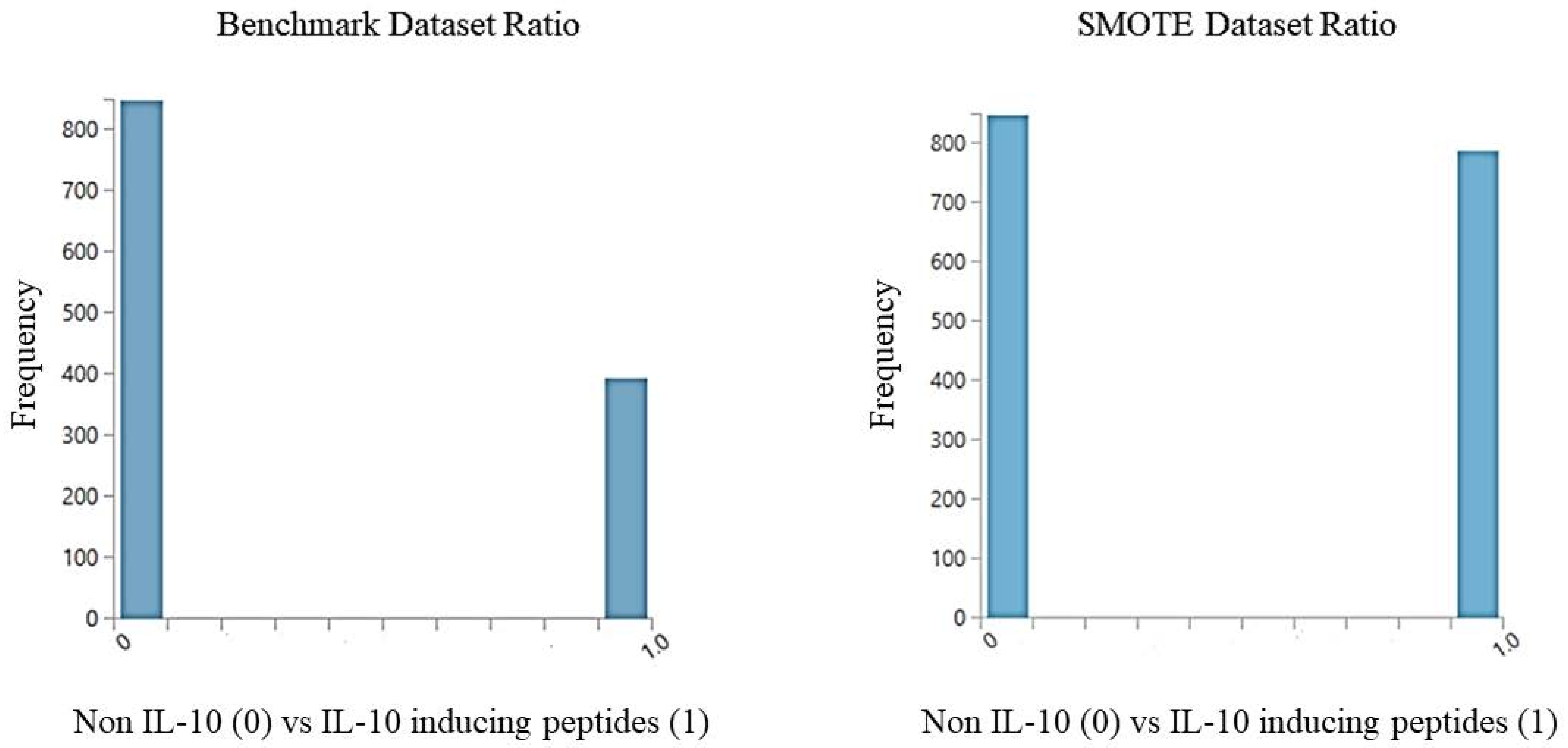

Benchmark Dataset Ratio
Frequency
800
700
600
500
400
300
200
100
0
0
1.0
Non IL-10 (0) vs IL-10 inducing peptides (1)
SMOTE Dataset Ratio
Frequency
800
700
600
500
400
300
200
100
0
0
1.0
Non IL-10 (0) vs IL-10 inducing peptides (1)
