# Supplementary material for: ILeukin10Pred: A Computational Approach for Predicting IL-10-Inducing Immunosuppressive Peptides Using Combinations of Amino Acid Global Features
Source: Biology (Basel). 2021 Dec 21;11(1):5. doi: 10.3390/biology11010005 (PMC8773200; doi:10.3390/biology11010005)
Supplement: Supplementary file 1 [file biology-11-00005-s001.zip › Figure S2.pdf]

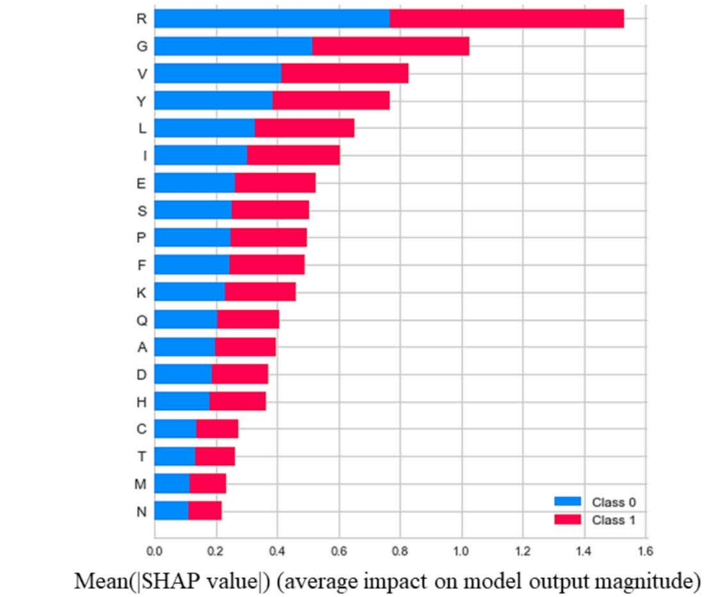


**Figure S2a** : Amino acid composition

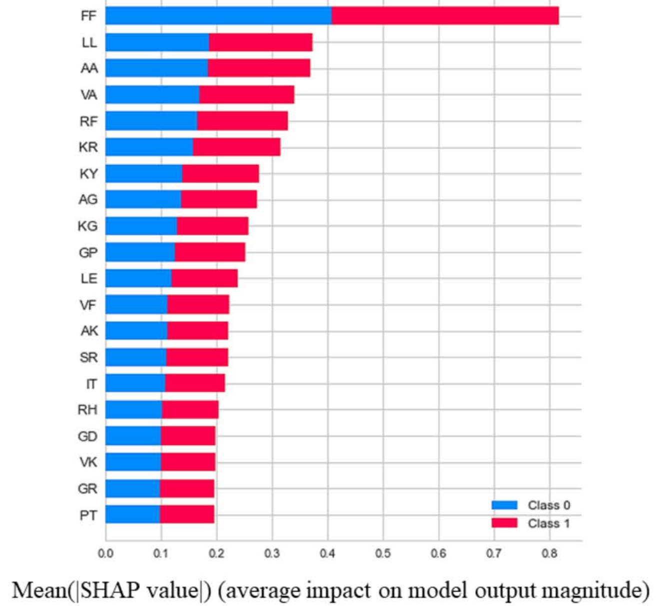


**Figure S2b**: Dipeptide composition

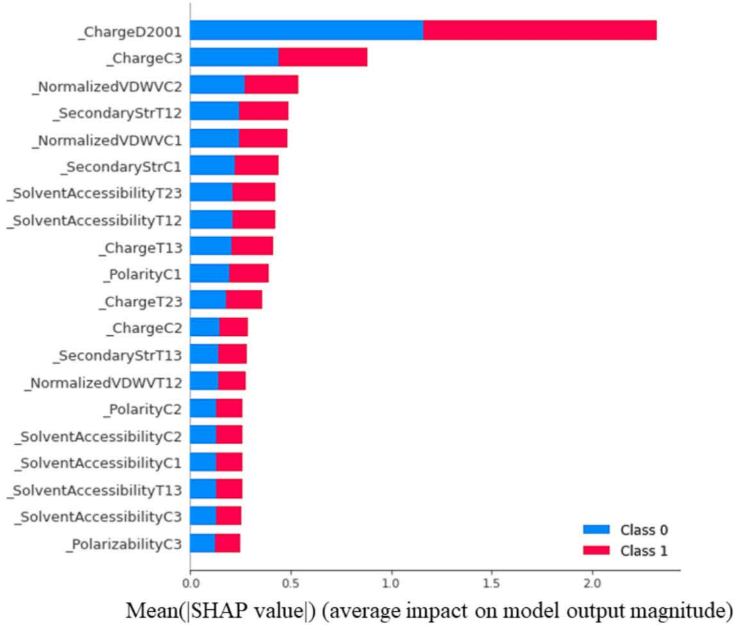


**Figure S2c** : CTD

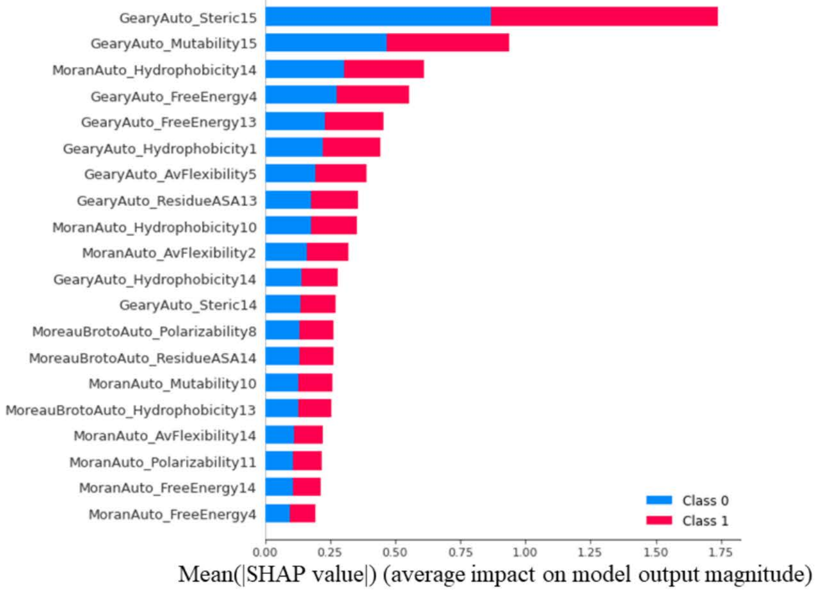


**Figure S2d**: AutoC

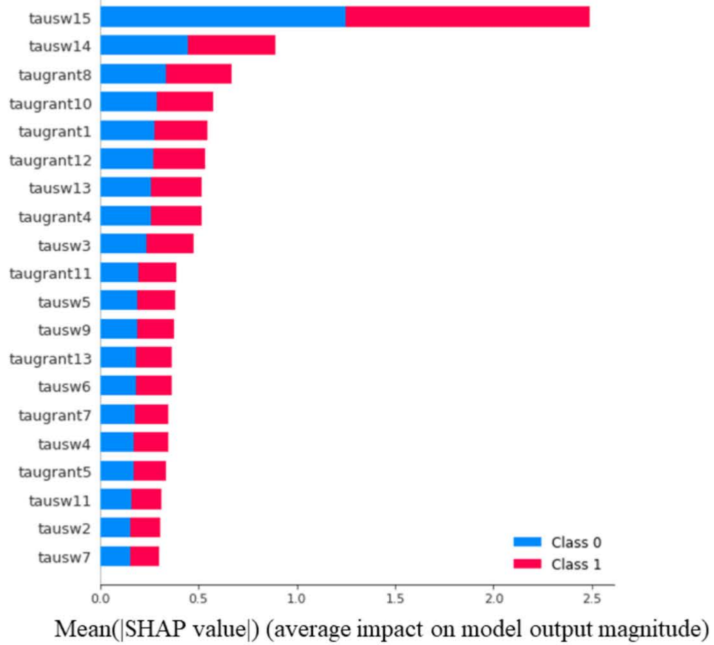


**Figure S2e**: SOC

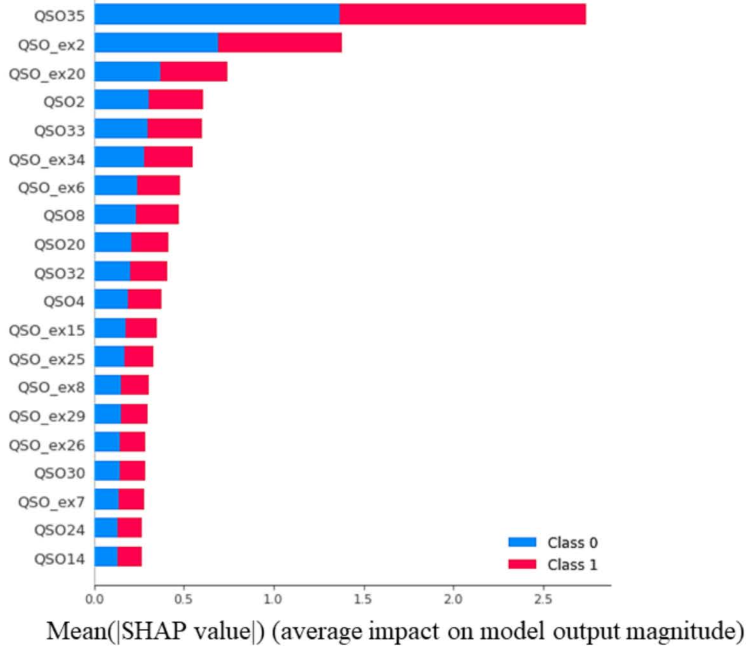


**Figure S2e**:QSO

**Figure S2**: Model interpretation based on SHapley Additive exPlanations (SHAP) for single feature types.
