# Supplementary material for: ILeukin10Pred: A Computational Approach for Predicting IL-10-Inducing Immunosuppressive Peptides Using Combinations of Amino Acid Global Features
Source: Biology (Basel). 2021 Dec 21;11(1):5. doi: 10.3390/biology11010005 (PMC8773200; doi:10.3390/biology11010005)
Supplement: Supplementary file 1 [file biology-11-00005-s001.zip › Supplementary Table S1.pdf]

Supplementary Table S1

Performances of machine learning models based on unselected features of all single-feature types for the benchmark training and test datasets. Values shown are the mean ± standard. deviation for the training dataset

Training set									
**Feature**	ETC			CatBoost			LGBM		
	Acc. %	AUC	MCC	Acc. %	AUC	MCC	Acc. %	AUC	MCC
**AAC**	58.6 ± 0.007	0.621 ± 0.010	0.254 ± 0.021	58.9 ±0.013	0.624 ±0.013	0.254 ±0.035	59.1 ±0.011	0.627 ±0.014	0.258 ±0.033
**DPC**	81.0 ±0.020	0.892 ±0.009	0.621 ±0.041	82.6 ±0.021	0.896 ±0.025	0.653 ±0.046	81.9 ±0.023	0.897 ±0.018	0.639 ±0.047
**CTD**	68.3 ±0.007	0.754 ±0.012	0.384 ±0.026	69.0 ±0.008	0.766 ±0.015	0.390 ±0.019	69.2 ±0.008	0.777 ±0.011	0.410 ± 0.017
**AutoC**	82.7 ±0.026	0.906 ±0.022	0.658 ±0.0052	83.9 ±0.028	0.900 ±0.024	0.679 ±0.058	82.9 ±0.023	0.901 ± 0.026	0.659 ±0.047
**QSO**	82.1 ±0.018	0.895 ±0.024	0.645 ±0.037	81.2 ±0.024	0.881 ±0.027	0.626 ±0.050	79.9 ±0.021	0.869 ±0.023	0.599 ±0.042
**SOC**	82.2 ±0.015	0.892 ±0.013	0.645 ±0.032	81.5 ±0.017	0.885 ±0.025	0.636 ±0.034	79.7 ±0.032	0.872 ±0.025	0.593 ±0.064
**Test dataset**									
**Feature**	ETC			CatBoost			LGBM		
	Acc. %	AUC	MCC	Acc. %	AUC	MCC	Acc. %	AUC	MCC
**AAC**	60.4	0.644	0.308	60.1	0.638	0.301	60.1	0.638	0.301
**DPC**	84.5	0.916	0.695	81.4	0.897	0.634	84.2	0.892	0.686
**CTD**	71.6	0.785	0.434	73.1	0.790	0.466	70.1	78.4	0.450
**AutoC**	83.2	0.921	0.674	86.2	0.913	0.728	83.8	0.909	0.678
**QSO**	85.9	0.936	0.724	85.1	0.925	0.705	86.3	0.924	0.727
**SOC**	84.4	0.918	0.697	85.1	0919	0.705	81.4	0.889	0.634
