# Supplementary material for: ILeukin10Pred: A Computational Approach for Predicting IL-10-Inducing Immunosuppressive Peptides Using Combinations of Amino Acid Global Features
Source: Biology (Basel). 2021 Dec 21;11(1):5. doi: 10.3390/biology11010005 (PMC8773200; doi:10.3390/biology11010005)
Supplement: Supplementary file 1 [file biology-11-00005-s001.zip › Supplementary Table S2.pdf]

Supplementary Table S2

Performances based on various hybrid feature combinations on the benchmark training and test datasets.

**Training set**									
**Feature**	ETC			CatBoost			LGBM		
	Acc. %	AUC	MCC	Acc. %	AUC	MCC	Acc. %	AUC	MCC
AAC+DPC	86.3	0.934	0.730	85.9	0.922	0.719	85.7	0.927	0.714
CTD+AutoC	84.1	0.912	0.683	86.2	0.908	0.710	85.1	0.913	0.703
QSO+SOC	86.4	0.923	0.729	84.7	0.916	0.695	84.8	0.911	0.697
AAC+DPC+CTD	85.7	0.938	0.718	84.9	0.920	0.697	86.0	0.919	0.720
QSO+SOC+AutoC	83.8	0.916	0.679	84.7	0.912	0.694	84.5	0.919	0.689
**Test dataset**									
**Feature**	ETC			CatBoost			LGBM		
	Acc. %	AUC	MCC	Acc. %	AUC	MCC	Acc. %	AUC	MCC
AAC+DPC	87.2	0.946	0.751	85.9	0.922	0.719	84.4	0.926	0.715
CTD+AutoC	83.5	0.927	0.681	85.3	0.924	0.710	86.5	0.931	0.735
QSO+SOC	86.6	0.934	0.738	86.6	0.925	0.736	85.3	0.918	0.710
AAC+DPC+CTD	86.5	0.944	0.737	86.2	0.923	0.732	85.1	0.921	0.707
QSO+SOC+AutoC	84.4	0.930	0.700	85.7	0.922	0.714	85.1	0.926	0.703
