# Supplementary material for: ILeukin10Pred: A Computational Approach for Predicting IL-10-Inducing Immunosuppressive Peptides Using Combinations of Amino Acid Global Features
Source: Biology (Basel). 2021 Dec 21;11(1):5. doi: 10.3390/biology11010005 (PMC8773200; doi:10.3390/biology11010005)
Supplement: Supplementary file 1 [file biology-11-00005-s001.zip › Supplementary Table S3.pdf]

Supplementary Table S3

Performances of machine learning models based on unselected features on the hybrid dataset (Combinations of all feature types) for the benchmark training and test datasets. Values shown are the mean ± standard. deviation for the training dataset.

Training dataset						
**Model**	Accuracy	AUC	Recall/Sensitivity	Specificity	Precision	MCC
**ETC**	83.9±0.011	0.900±0.018	0.774±0.017	0.898±0.037	0.878±0.036	0.681±0.026
**LGBM**	82.4±0.016	0.882±0.025	0.784±0.016	0.862±0.034	0.843±0.032	0.650±0.034
**CatBoost**	82.4±0.022	0.890±0.024	0.758±0.033	0.886±0.029	0.843±0.029	0.651±0.044

Test dataset						
**Model**	Accuracy	AUC	Recall/Sensitivity	Specificity	Precision	MCC
**ETC**	83.5	0.910	0.721	0.941	0.919	0.682
**LGBM**	83.2	0.891	0.753	0.906	0.881	0.669
**CatBoost**	82.9	0.907	0.721	0.929	0.904	0.667
